# Prognostic Relationship Between the ABO Blood Groups and Metastatic Gastric Cancer

**DOI:** 10.7759/cureus.34837

**Published:** 2023-02-10

**Authors:** Elif Tugba Tuncel, Engin Kut

**Affiliations:** 1 Gastroenterology, Manisa State Hospital, Manisa, TUR; 2 Medical Oncology, Manisa State Hospital, Manisa, TUR

**Keywords:** tumor location, histopatology, prognosis, advanced gastric cancer, abo blood groups

## Abstract

Aim: Gastric cancer is one of the most common malignant tumors of the digestive system and has a poor prognosis. Since recurrence and distant metastasis are common in gastric cancer, it is important to use practical and reliable prognostic parameters. In this study, the prognostic relationship between the ABO blood groups and metastatic gastric cancer was investigated.

Method and Material: Data were collected by retrospectively scanning the files of 225 patients who were followed up with the diagnosis of metastatic gastric cancer in 2010-2022. The patients’ demographic data (age, gender), tumor histopathology, tumor location, and ABO and Rh blood groups were evaluated.

Results: Of the patients, 138 (61.3%) were male and 87 (38.7%) were female. According to the distribution of the ABO system, blood group A was present in 109 (48.4%) patients, B in 33 (14.7%), AB in 20 (8.9%), and O in 63 (28%). Signet ring cell carcinoma, antrum tumor localization, and distant metastasis were more common in blood groups A and O. According to both the univariate and multivariate analyses, overall survival (OS) was statistically worse in patients with signet ring cell carcinoma and peritoneal metastasis (p < 0.05). The OS rate was the worst in blood group A and best in blood groups AB and B.

Conclusion: In this study, blood group A presented as both a risk factor and a poor prognostic factor in the development of metastatic gastric cancer. In addition, signet ring cell histopathology and presence of metastasis were found to be more common in patients with blood group A and associated with a poor prognosis. Blood groups are inexpensive, easily available, and reliable parameters that can provide an idea about both prognosis and survival in gastric cancer. Therefore, they can serve as a guide for clinicians in the follow-up and evaluation of the prognosis of these patients.

## Introduction

Gastric cancer is the fourth most common malignancy across the world and the third most common cause of cancer-related death [1-3]. In 95% of cases, gastric cancer develops from epithelial cells. Stromal tumors and lymphomas that constitute the remaining cases originate from non-epithelial cells. Gastric cancer is divided into cardia and non-cardia according to the anatomical localization and intestinal, diffuse, and mixed type according to the histological type. The five-year survival rate is 90% in patients with early gastric cancer confined to the mucosa but falls below 40% in advanced stages [4-6]. Gastric cancer has a poor overall prognosis as the diagnosis is generally delayed. Knowing risk factors associated with gastric cancer is important in terms of preventing the disease and evaluating the prognosis. The ABO blood groups are one of the most stable genetic factors routinely included in the preoperative hematological examination. ABO blood group antigens are expressed on malignant or non-malignant cells and responsible for the initiation and spread of malignancies and the systemic inflammatory response. The ABO blood groups may increase the incidence of certain cancer types, and therefore can predict prognosis [7-9]. The relationship of the ABO blood groups, which is an important risk factor in the development of gastric cancer, with cancer prognosis is still being investigated. Since recurrence is common, there is a need for individualized prognostic factors for the evaluation of post-treatment survival and prognosis. This study aimed to investigate the correlation of the metastatic gastric cancer and ABO blood groups with histopathology, tumor localization, and overall survival (OS) and determine the prognostic significance of blood groups in gastric cancer.

## Materials and methods

Data were retrospectively collected by screening the files of 225 patients with metastatic gastric cancer followed up at Manisa City Hospital between 2010 and 2021. Patients with clinicopathologically confirmed primary gastric cancer without any other synchronous malignancy were included in the study. The patients’ demographic data (age, gender), tumor histopathology, presence of metastasis, tumor localization, ABO blood group, and Rh group were evaluated. OS was defined as the time from the diagnosis to death and evaluated with the multivariate and univariate analyses. OS time of 5 years was evaluated. History of surgery, tumor stage, lymphovascular invasion, and distant organ metastasis status were evaluated. Those under 18 years of age and those with a history of other solid or hematological malignancies were excluded from the study.

Statistical analysis

Descriptive statistics were presented as numbers and percentages for categorical variables, median with minimum and maximum values and mean ± standard deviation values for numerical variables. Visual (histogram) and analytical (Kolmogorov-Smirnov/Shapiro-Wilk test) methods were used to determine the distribution of variables. Normally distributed parameters were compared using Student’s t-test and non-normally distributed variables using the Mann-Whitney U test. Independent groups were compared with the chi-square or Fisher’s exact test. Survival curves were obtained with the Kaplan-Meier analysis. Significant parameters in the univariate analysis were further evaluated using the Cox regression. A p value of <0.05 was considered significant in all the statistics.

## Results

Of the patients, 138 (61.3%) were male and 87 (38.7%) were female. The mean age was 62 (range 27-83) years. The median follow-up time was 10 months, and the median survival time was 13 months [95% confidence interval (CI): 8.3-11.2]. According to the distribution of the ABO system, blood group A was present in 109 (48.4%) patients, B in 33 (14.7%), AB in 20 (8.9%), and O in 63 (28%). In addition, 196 (87.1%) patients were Rh (+), and the remaining 29 (12.9%) were Rh (-). The distribution of the ABO blood groups was previously reported as A, 41%; O, 47%; B, 9%; and AB, 3% globally and A, 42.8%; 0, 32%; B, 16%; AB, 8%; and Rh (+), 85.8% for Turkey [10]. There were 92 (40.9%) patients with signet ring cell carcinoma and 133 (59.1%) with non-signet ring cell carcinoma. Liver metastasis was present in 99 (44%) patients, lung metastasis in 65 (28.9%), peritoneal metastasis in 109 (48.4%), bone metastasis in 81 (36%), and lymph node metastasis in 106 (47.1%). The mean OS time was 6.47 months (95% CI: 5.06-7.83) for blood group A, 17.31 months (95% CI: 15.45-18.03) for blood group AB, 14.56 months (CI: 10.16-19.43) for blood group B, and 11.77 months (8.55-12.99) for blood group O (Table 1).

**Table 1:** Demographic and clinical characteristics of the patients according to their blood groups. ECOG, Eastern Cooperative Oncology Group performance score

	A	AB	B	O	p value
Female	45 (51.7%)	9 (10.3%)	13 (14.9%)	20(%23)	0.58
Male	64 (46.4%)	11 (8%)	20 (14.5%)	43 (31.2%)	
Liver metastasis	47 (47.1%)	11 (11.1%)	14 (14.1	27 (27.3%)	0.781
Lung metastasis	35 (53.19	7 (10.8%)	7 (10.8%)	16 (24.6%)	0.525
Peritoneal metastasis	59 (54.1%)	2 (1.8%)	14 (12.8%)	34 (31.2%)	0.002
Lymph node involvement	49 (46.2%)	12 (11.3%)	11(10.4%)	34 (32.1%)	0.155
ECOG ≥ 2	36 (45.69	7 (8.9%)	8 (10.19	28 (35.4%)	0.228
ECOG < 2	73 (50%)	13 (8.9%)	25 (17.1%)	35 (24%)	
Tumor localization					0.228
Antrum	46 (42%)	5 (13%)	11 (33%)	21 (33%)	
Corpus	36 (33%)	22 (59%)	15 (45%)	30 (47%)	
Cardia	27 (24%)	10 (27%)	7 (21%)	12 (19%)	
Signet ring cell carcinoma	60 (65.2%)	5 (5.4%)	11 (12%)	16 (17.4%)	0.01
Non-signet ring cell carcinoma	49 (36.8%)	15 (11.3%)	22 (16.5%)	47 (35.3%)	

**Figure 1:**
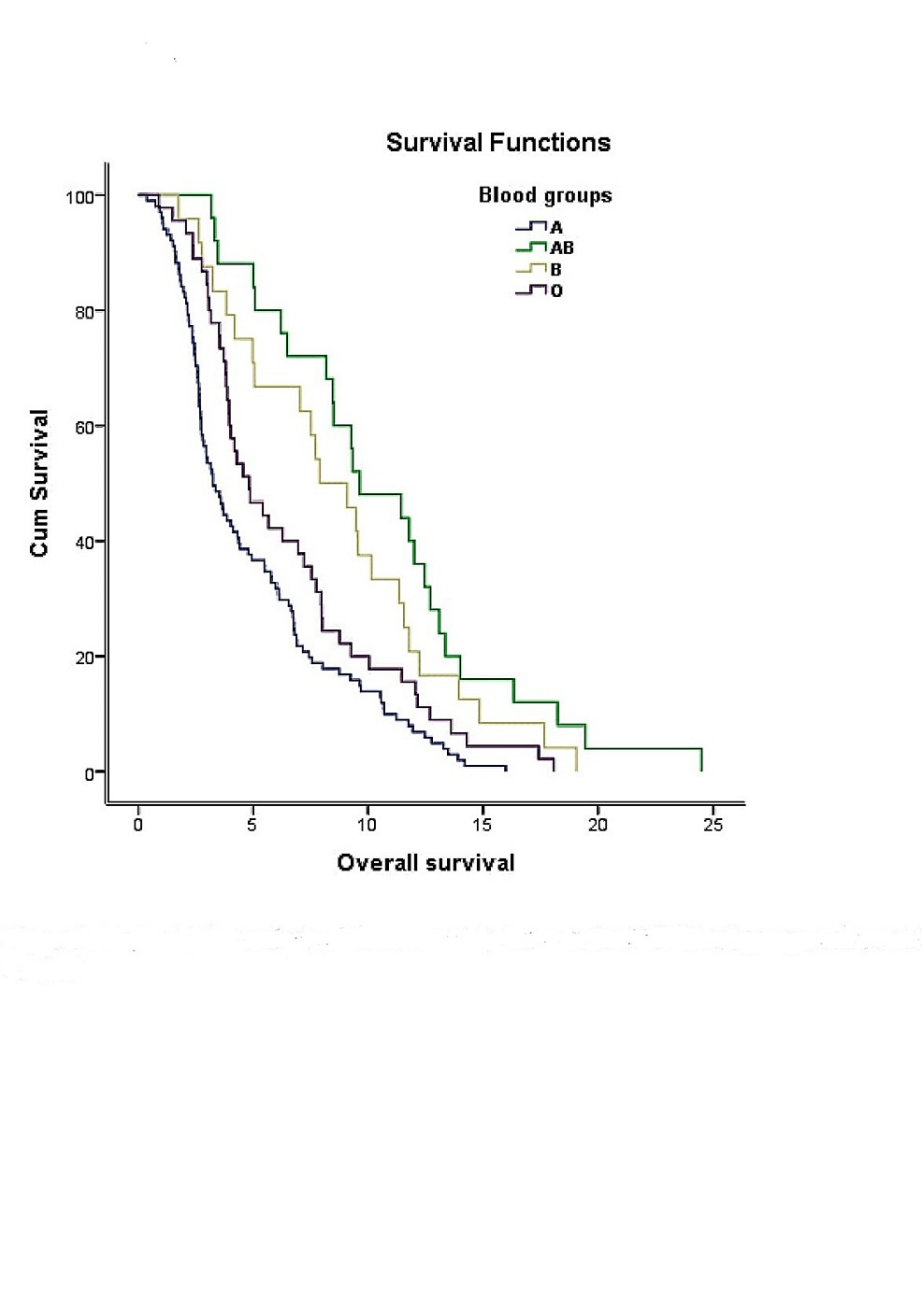
Kaplan‐Meier analysis of the relationship between the ABO blood groups and overall survival (5 year OS). OS, overall survival

 Signet ring cell gastric carcinoma was most common in blood group A and least common in blood group AB, and this was at a statistically significant level. There was no statistically significant difference between the blood groups in relation to the incidence of non-signet ring cell carcinoma. The patients with signet ring cell carcinoma, distant metastasis, and antrum tumor localization were found at a higher rate in blood groups A and O. The results of the univariate analysis showed that age, gender, Rh group, tumor localization, and presence of lymph node, lung, and liver metastases did not affect OS. However, according to both the univariate and multivariate analyses, OS was statistically worse in the patients with signet ring cell histopathology, peritoneal and bone metastases, and Eastern Cooperative Oncology Group performance score < 2 (p < 0.05) (Table 2). Five year OS and prognosis were worst in blood group A and best in blood groups AB and B (Figure 1).

**Table 2:** Univariate and multivariate analyses of overall survival. OS, overall survival

	Univariate		Multivariate	
	P	Hazard ratio	p value	Hazard ratio
Age	0.44	1.005 (0.99-1.017)		
Gender	0.557	1.087 (0.823-1.435)		
Rh group	0.108	1.390 (0.930-2.076)		
Signet ring cell	<0,001	1.788 (1.348-2.371)	<0,0001	-0.509 (0.498-0.787)
Tumor localization	0.422	1.007 (0.937-1.081)		
ECOG	0.001	1.611 (1.203-2.158)	<0.0001	-0.539 (0.418-0.777)
Peritoneal metastasis	0.002	1.595 (2.185-2.059)	0.166	-0.809 (0.600-1.091)
Liver metastasis	0,097	1.264 (0.959-1.605)		
Lung metastasis	0,788	1.042 (0.771-1.408)		
Bone metastasis	0,033	1.365 (1.025-1.817)		
Lymph node involvement	0,311	1.152 (0.87-1.5369)		

## Discussion

The ABO blood groups have an important place in hematology, transfusion, and transplantation clinics. Blood group antigens, along with erythrocytes, are expressed on gastrointestinal and bronchopulmonary epithelial cells. Therefore, they play an important role in the development of many cardiovascular, infectious, and neoplastic diseases. In recent studies, blood groups were found to be an important risk factor for the development of gastric cancer [11]. Our results also revealed that blood group A was both a risk and prognostic factor in metastatic gastric cancer, being detected more frequently among women and patients with antrum tumor localization. In contrast, the risk of gastric cancer was lower in the patients with blood group AB.

 Blood group carbohydrates are expressed on the cancer cell surface function as cell adhesion molecules. These adhesion molecules are important mediators of chronic inflammation and immune cell response. ABO blood group antigens affect tumor formation, spread, and prognosis by modulating von Willebrand factor. The loss of ABO antigens (A, B, and H) reduces apoptosis, promotes immune escape, and cellular adhesion. Tumor immune response is decreased as a result of the reduced ability to recognize and attack tumor cells in individuals with blood group A; thus, the metastatic potential of tumors increases and OS decreases [12-13].

 Gastric carcinoma cells and blood group A produce immunologically similar antigens. This carcinoma develops more easily in individuals with blood groups A and AB because they do not have antibodies against A. This antigen provides a protective effect by preventing tumor growth and spread in individuals with blood group O [14-15]. In a study by Aird et al. in 1953, it was shown, for the first time, that blood group A was more common among patients with gastric cancer [16]. In a later study, Edgren et al. reported that blood group A was associated with the risk of gastric cancer, and blood group O was associated with the risk of peptic ulcer [17-18]. In a retrospective analysis of 1,412 patients with gastric cancer that underwent radical gastrectomy, Xu et al. determined blood group AB as a good prognostic factor and blood group A as a poor prognostic factor [19]. In addition, Ziaul Haque et al. found gastric cancer to be more common and associated more with poorly differentiated adenocarcinoma in those with blood group B [20]. In a study by Hao et al., individuals with blood group A were reported to be more likely to develop gastric cancer, while blood group O resulted in a relatively better prognosis [21]. Wang et al. showed that the risk of *Helicobacter pylori* infection and gastric cancer increased in individuals with blood group A [22], and Nakao et al. detected a higher risk of gastric cancer, atrophic gastritis, and *H. pylori* infection in the AA genotype [23], revealing that blood group antigens could affect prognosis and survival in gastric cancer by binding to the gastric mucosa of *H. pylori*. In a case control study conducted by Song et al. with 3,245 patients with gastric cancer and 1,700 controls, the relationship between the ABO genotype and gastric cancer risk, and the risk of gastric cancer was observed to be higher among the women with the AA and AO genotypes compared to the men. The authors concluded that the AO genotype was significantly associated with the common type of gastric cancer [24]. In the current study, the incidence of signet ring cell histopathology and peritoneal metastasis was higher in the patients with blood group A, and this blood group was found to be an independent poor prognostic factor. There is no other study in the literature showing the relationship between blood group, histopathology, and organ metastasis. Therefore, we consider that our results can contribute to the literature. However, this study also had certain limitations, including the retrospective and single-center design, which also resulted in a small number of patients. There is a need for multicenter studies with larger populations. We also consider that further studies should be conducted to investigate the relationship of the ABO blood groups with the main risk factors of gastric cancer, such as pernicious anemia, atrophic gastritis, gastric pH, pepsinogen level, and *H. pylori* infection.

## Conclusions

Blood groups have both predictive and prognostic value in metastatic gastric cancer. We determined that the risk of gastric cancer development was higher and the prognosis was worse in the patients with blood group A, while the prognosis and survival were better in those with blood group AB. Signet ring cell pathology and antrum localization are more common in those with A blood group. Signet ring cell histopathology was found to be an important factor affecting survival and prognosis. There are few studies evaluating the prognostic relationship between blood groups and histopathology in gastric cancers. Therefore, we think that our results will contribute to the literature. When assessing an individual patient’s risk, the ABO blood groups should be considered together with other risk factors. As a simple, easily available, inexpensive, and practical method, blood groups can be used as a prognostic factor in routine clinical practice in patients with gastric cancer.
